# Transcriptome Meta-Analysis Deciphers a Dysregulation in Immune Response-Associated Gene Signatures during Sepsis

**DOI:** 10.3390/genes10121005

**Published:** 2019-12-04

**Authors:** Shaniya Ahmad, Prithvi Singh, Archana Sharma, Shweta Arora, Nitesh Shriwash, Arshad Husain Rahmani, Saleh A. Almatroodi, Kailash Manda, Ravins Dohare, Mansoor Ali Syed

**Affiliations:** 1 Translational Research Lab, Department of Biotechnology, Faculty of Natural Sciences, Jamia Millia Islamia, New Delhi 110025, India; shaniya169053@st.jmi.ac.in (S.A.); archanagmsharma@gmail.com (A.S.); shweta169213@st.jmi.ac.in (S.A.); 2 Centre for Interdisciplinary Research in Basic Sciences, Jamia Millia Islamia, New Delhi 110025, India; prithvi.mastermind@gmail.com; 3 Department of Computer Science, Faculty of Natural Sciences, Jamia Millia Islamia, New Delhi 110025, India; niteshkumar20sept@gmail.com; 4 Department of Medical Laboratories, College of Applied Medical Sciences, Qassim University, Buraidah 51452, Saudi Arabia; ah.rahmani@qu.edu.sa (A.H.R.); smtrody@qu.edu.sa (S.A.A.); 5 Institute of Nuclear Medicine and Applied Sciences, Defence Research Development Organization, New Delhi 110054, India; kailashmanda@gmail.com

**Keywords:** sepsis, meta-analysis, DEG, PPI

## Abstract

Sepsis is a life-threatening disease induced by a systemic inflammatory response, which leads to organ dysfunction and mortality. In sepsis, the host immune response is depressed and unable to cope with infection; no drug is currently available to treat this. The lungs are frequently the starting point for sepsis. This study aimed to identify potential genes for diagnostics and therapeutic purposes in sepsis by a comprehensive bioinformatics analysis. Our criteria are to unravel sepsis-associated signature genes from gene expression datasets. Differentially expressed genes (DEGs) were identified from samples of sepsis patients using a meta-analysis and then further subjected to functional enrichment and protein‒protein interaction (PPI) network analysis for examining their potential functions. Finally, the expression of the topmost upregulated genes (*ARG1*, *IL1R2*, *ELANE*, *MMP9*) was quantified by reverse transcriptase-PCR (RT-PCR), and myeloperoxidase (*MPO*) expression was confirmed by immunohistochemistry (IHC) staining in the lungs of a well-established sepsis mouse model. We found that all the four genes were upregulated in semiquantitative RT-PCR studies; however, *MMP9* showed a nonsignificant increase in expression. *MPO* staining showed strong immunoreactivity in sepsis as compared to the control. This study demonstrates the role of significant and widespread immune activation (*IL1R2*, *MMP9*), along with oxidative stress (*ARG1*) and the recruitment of neutrophils, in sepsis (*ELANE*, *MPO*).

## 1. Introduction

Sepsis is a clinically heterogeneous and biologically complex disease characterized by the improper response of the immune system due to an infection caused by bacteria, viruses, fungi, or parasites, leading to organ dysfunction [1]. Globally, 30 million people are affected by this life-threatening disease each year, of which 6 million deaths have been reported annually [2,3]. In recent times, sepsis incidence rates have increased rapidly and the World Health Organization (WHO) has declared it as a key healthcare priority for the coming decade. Despite the best possible therapies such as antimicrobial therapy, hemodynamic resuscitation, and supportive therapy (lung-protective ventilation, use of sedatives, nutrition management) [4], this disease is evolving over time and becoming a critical issue for clinicians and researchers. The major cause of sepsis is a lack of knowledge about the pathophysiological and biochemical mechanisms behind the perturbation of the host immune response. This reflects the patient’s risk and often involves a delay in diagnosis. The pathogenesis of sepsis on a genetic basis is underappreciated, but death from this acute condition is more heritable than cancer [5]. In the pathogenesis of sepsis, genetic factors play an important role; however, the mRNAs associated with sepsis still need to be explored.

A high-throughput technology such as microarray is a tool of expression analysis that provides information on the genetic contribution to sepsis and other diseases [6]. Datasets obtained through this have been used to detect the differentially expressed genes (DEGs) in sepsis and healthy individuals to explore its pathogenesis. For instance, the microarray data obtained by Tang et al. [7] were analyzed by Qiao et al. [8] to identify the DEGs associated with different pathways in sepsis pathogenesis. Another study from Wang et al. [9] analyzed potential biomarkers of severe sepsis with multiple organ failure using a microarray dataset and found that lung failure sepsis had the highest number of DEGs. These collaborative studies established on transcription profiling analysis can provide information to guide future research. This high-throughput technology assesses various mRNA levels of diverse genes simultaneously in a highly cost-effective manner and helps us to understand and analyze global genomic patterns of diverse diseases [10]. Despite these advantages, the genes distinguished in one study are often not distinguished in other studies [11]. To improve the reliability of results, integrating information from multiple studies has been reported [6]. The meta-analysis approach merges information from different datasets that share a molecular mechanism of disease. This helps to combat the inconsistency in results which may have arisen due to differences in the microarray platforms, sample source, and analysis techniques [11]. This approach thoroughly studies the available data in a relatively inexpensive manner. Therefore, an accurate estimation of gene expression differentials can be obtained, and the heterogeneity of a comprehensive estimate can be accessed through it. This approach has been utilized in various complex diseases to identify the key genes. Our study criteria whirl around the establishment of crucial genes associated with sepsis. Uncovering the genes responsible for the disease is a prerequisite for the prompt identification and diagnosis of a disease. We conducted our study to determine the role of signature genes associated with sepsis, based on the GSE13904 [12] and GSE54514 [13] datasets retrieved from the Gene Expression Omnibus (GEO) database [14]. DEGs were identified using a meta-analysis approach. The functions of these potential DEGs were analyzed using the Gene Ontology (GO) and pathway enrichment analysis which was conducted using DAVID (the Database for Annotation, Visualization and Integrated Discovery [15]. Subsequently, a protein‒protein interaction (PPI) network for functionally enriched DEGs was constructed using BioGRID (the Biological General Repository for Interaction Datasets) [16] and visualized in Cytoscape respectively. Finally, we utilized the reverse transcriptase-PCR and immunohistochemistry techniques to validate the few topmost upregulated genes. 

## 2. Materials and Methods

### 2.1. Sepsis-Associated Gene Expression Dataset Extraction

For microarray dataset selection, the GEO database was exhaustively searched. The criterion for selection process was based on the PRISMA (Preferred Reporting Items for Systematic Reviews and Meta-Analysis) guidelines published in 2009 [17]. Benchmarking for the selection of gene expression datasets was as follows: Organism—*Homo sapiens*, Study type—Expression profiling by array, and Attribute name—Tissue. GEO datasets possessing accession numbers GSE13904 and GSE54514 were selected from the National Center for Biotechnology Information (NCBI) GEO after a thorough search. For conducting the meta-analysis, necessary information was extracted from these two datasets. Here we considered both sepsis and septic shock samples as sepsis cases. Series matrix expression files of both these datasets were extracted for further analysis. Every probe in the expression file was allocated to its respective HGNC (HUGO Gene Nomenclature Committee) [18] gene symbol(s). To achieve this, numerous databases and tools were used, such as Synergizer [19], bioDBnet:db2db conversion [20], gprofiler ID converter [21], AbIDconvert [22], and GEO2R. Duplicate gene symbols mapping to multiple probe IDs were eliminated by averaging their relative expression vaules [23]. Sepsis-infected CLP mice model datasets possessing accession numbers GSE24357 [24] and GSE15379 [25] were also extracted from GEO. The CLP sample probe IDs were mapped to their respective genes using GEO2R.

### 2.2. Meta-Analysis and DEGs Screening

An unpaired *t*-test with Welch’s correction was applied for all genes within each study [26] to compare the gene expression vaules in sepsis samples with those of control subjects. The *t*-test for unpaired data and both for an equal and unequal variance can be computed as follows:

(1) $$t=\frac{{\overset{´}{X}}_{1}-{\overset{´}{X}}_{2}}{\sqrt{\frac{s_{1}^{2}}{N_{1}}+\frac{s_{2}^{2}}{N_{2}}}},$$

where ${\overset{´}{X}}_{1}$ and ${\overset{´}{X}}_{2}$ are the means, $s_{1}^{2}$ and $s_{2}^{2}$ are the variances, and $N_{1}$ and $N_{2}$ are the sizes of the two groups of the samples. A *p*-vaule was returned for each gene during the *t*-test. R software v. 3.6.1 was used to conduct this *t*-test on gene profiles obtained from the retrieved datasets.

Then we conducted a meta-analysis by combining *p*-vaules according to the Fisher’s combined probability test method [27] in R using the formula

(2) $$X_{2k}^{2}=-2\sum_{i=1}^{k}ln\left(p_{i}\right),$$

where *p_i_* is the *p*-vaule, *k* is the number of tests being combined and *2k* is the degrees of freedom. The *p* − vaules were adjusted using the approach of false discovery rate (FDR), as given in the Benjamini‒Hochberg (BH) method [28].

At this stage, we calculated the fold change (FC) vaule for each gene to be used for filtering purposes. FC is a measure that describes how much the expression level of a gene changes over two different samples (conditions) or groups. The FC for linear data can be calculated as follows:

(3) $$FC_{i}=log_{2}\frac{{\overset{´}{y}}_{i}}{{\overset{´}{x}}_{i}}\;\mathrm{or}\;log_{2}{\overset{´}{y}}_{i}-log_{2}{\overset{´}{x}}_{i},$$

where ${\overset{´}{x}}_{i}$ and ${\overset{´}{y}}_{i}$ are the means of the gene expression profiles of the control group and sepsis group, respectively. In this case, where the gene expression data are already in *log_2_*-transformed form, FC can be computed as follows:

(4) $$FC_{i}=\frac{{\overset{´}{y}}_{i}}{{\overset{´}{x}}_{i}}\;\mathrm{or}\;{\overset{´}{y}}_{i}-{\overset{´}{x}}_{i}.$$

The two log-fold changes obtained for each gene were averaged to produce a single log-FC. The DEGs between sepsis and normal healthy individuals were selected based on a certain criterion, i.e., *p*-vaules < 0.05 and FC > 2. To find the genes that are differentially expressed in a specific group, we checked for superimposed DEGs in sepsis day1 samples and sepsis day3 samples. For identifying overlapping genes between both groups, we used Venny 2.1.0.

### 2.3. Functional and Pathway Enrichment Analysis 

After a meta-analysis, the biological implications of identified DEGs is important to understand, so pathway and functional enrichment analysis was performed. We carried out GO and pathway enrichment analysis (Kyoto Encyclopedia of Genes and Genomes, KEGG), using DAVID v6.8 (David.abcc.ncifcrf.gov) [29], with a significance of *p*- vaule < 0.05.

### 2.4. PPI Network Construction and Analysis

A PPI network was created using Cytoscape v3.7.2 [30] to further understand and predict the biological activity of the identified DEGs based on GO and KEGG enrichment analysis. The DEGs’ encoding proteins and their interacting partners were computed from the BioGRID database [31] for PPI network construction. This PPI network was subsequently visualized in Cytoscape. A box- and -whisker plot is a very informative tool that helps us to gain an insight into the distribution of data. The *box plot* function in R was used to create the box- and -whisker plot.

### 2.5. Animal Model

In total, six C57BL/6 mice (six weeks old, 20–25 g) were obtained from the Animal House Facility of Defence Research Development Organization (DRDO)‒Institute of Nuclear Medicine and Allied Science (INMAS), New Delhi. The study protocol was approved by the Institutional Animal Ethics Committee (IAEC) of DRDO-INMAS (INM/IAEC/2018/25/ext). Animals were caged under stable conditions (temperature: 21 ± 2,12 h light/dark cycle and humidity: 50–60). Animals had access to food and water *ad libitum*. Experiments were done with the utmost care and according to the guidelines.

### 2.6. Experimental Protocol

Animals were divided into two groups: the Cecal Ligation and Puncture (CLP) group and a sham group (*n* = 3/group). CLP was performed according to the protocol followed by Das et al. [32]. For CLP group animals, the lower areas of the abdomen were shaved and disinfected, and an incision was made. After dissection, the cecum was ligated below the ileocecal valve, followed by through and through puncture using a 26-gauge needle. The cecum was then placed back in peritoneal cavity and the peritoneum was closed using absorbable suture 4.0 Chromic (Ethicon, New Jersey, NJ, USA lot no-B7002). The skin was closed using non-absorbable 4.0 silk suture (Ethicon, New Jersey, NJ, USA lot no-B7006) and then betadine was applied around the surgery area. Sham group animals underwent the same procedure except for the puncture and ligation. After surgery, animals were returned to their cages and provided with food and water *ad libitum*. After 24 h of treatment, the animals were sacrificed, and lung tissues were harvested and stored at −80 °C until RNA extraction and formalin-fixed for immunohistochemistry analysis.

### 2.7. Semiquantitative RT-PCR

Extraction of RNA was done from lung tissue using TRIZOL (Ambion, Carlsbad, CA, USA) in accordance with the manufacturer’s protocol. cDNA was synthesized using Bio-Rad’s (Hercules, CA, USA) iScript cDNA synthesis kit and amplified for Arginase 1 (*ARG1*), Interleukin 1 Receptor Type 2 (*IL1R2*), Matrix Metallopeptidase 9 (*MMP9*), and Elastase, Neutrophil Expressed (*ELANE*) using a PCR green master mix (Promega, Madison, WI, USA). Actin was used as an endogenous control gene for data normalization. The PCR thermocycling conditions for 35 cycles were as follows: Initial denaturation for 5 min at 95 °C, cycle with denaturation for 45 s at 95 °C, annealing for 1 min at (48.5 °C—*ARG1*), (63 °C—*IL1R2*), (54 °C—*MMP9* and *ELANE*). Primer extension for 1 min at 72 °C and final extension for 5 min at 72 °C PCR amplicons were run on a 1% agarose gel electrophoresis containing ethidium bromide (1 mg/mL). A Gel Doc EZ system (Bio-Rad) was used for gel picture visualization and the intensity of bands was quantified using ImageJ (Bethesda, Maryland, MD, USA) software. The results are relative to endogenous control actin expression. The sequences of the primer used were: *IL1R2*: GGTGCGGACAATGTTCATCTTG and GGGAACTGCTGGAGATCTCGGAGTG: Product size—239 bp, *ARG1*: CAGAAGAATGGAAGAG

TCAG and CAGATATGCAGGGAGTCACC: Product size—250 bp, Actin: fwd: CTGTCCCTGTATGCCTCTG Rev: ATGTCACGCACGATTTCC: Product size—220 bp. *MMP9*: fwd: CGTCGTGATCCCCACTTACT Rev: AACACACAGGGTTTGCCTTC: Product size—405 bp, *ELANE*: fwd: GGCTTTGACCCATCACACAACT: Rev: CGGCACATGTTAGTCACCAC.

### 2.8. Immunohistochemistry for MPO

Lung tissues were formalin-fixed and embedded in paraffin. Five-micrometer sections were cut and dewaxed with xylene, hydrated, and the antigen retrieved in a citrate buffer (pH: 6.00, 98 °C) for 20 min. Endogenous peroxidase activity was blocked by 3% H_2_O_2_ for 10 min. Subsequently, the sections were incubated with 5% bovine serum albumin for 30 min. *MPO* heavy chain goat polyclonal (Santa Cruz, CA, USA) antibody was added and incubated overnight at 4 °C in a humid chamber. Afterwards, the sections were washed and incubated with biotin-labeled rabbit anti-goat secondary antibody. The sections were washed again and then incubated with an avidin‒peroxidase complex (ImmunoCruz ABC kit, Santa Cruz). Slides were stained with 3, 3′ Diamobenzidine (DAB, ChemCruz) to prompt the *MPO* to be visualized and then counterstained with hematoxylin to dye the cell nucleus. Dehydration with alcohol series was done and then sections were placed in xylene for differentiation. Finally, the sections were mounted using a DPX mount and visualized under a microscope, and image quantification was done using ImageJ software (Bethesda, Maryland, MD, USA). 

### 2.9. Statistical Analysis

Data are represented as mean ± SEM. Results were analyzed by an unpaired *t*-test. Statistical significance was obtained when *p*-vaules were less than 0.05 and 0.0001 using GraphPad Prism 6 software (La Jolla, CA, USA).

## 3. Results

### 3.1. Sepsis-Associated Microarray Dataset Selection

**Figure 1:**
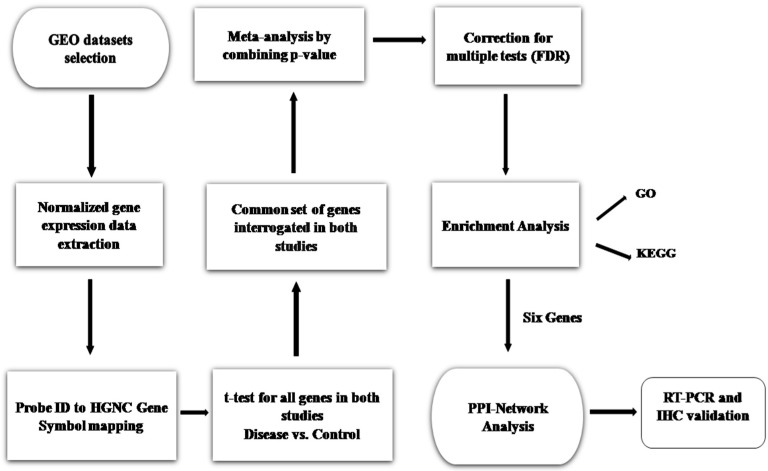
Proposed methodology workflow. Analysis of genes was performed by comparing sepsis with control. From these two datasets, the *p*-vaules were calculated and then combined to compute a single *p*-vaule per gene, adjusted for multiple testing (FDR). One hundred and forty-six genes with significant *p*-vaule < 0.05 and FC > 2 were considered as differentially expressed (upregulated) in sepsis. The 146 upregulated genes were subjected to enrichment analysis. A protein‒protein interaction (PPI) network of the identified differentially expressed genes (DEGs) based on the pathway and Gene Ontology (GO) term enrichment analysis was constructed. Thereafter, RT-PCR and immunohistochemistry (IHC) validation studies were performed.

The GEO datasets possessing accession numbers GSE13904 and GSE54514 contain a total of 275 human samples, of which 134 were case samples of day1, 87 of day3, and 54 control samples. The significant dossiers were extracted from exclusive studies such as accession number of GEO, sample source, number of cases and controls, and gene expression, platform, and profile (Table 1). To detect DEGs, normalized expression data were obtained from GEO and an analysis was done based on two criteria, FC and *p*-vaule (Figure 1). A comparison was done between sepsis and control for individual genes and the *p*-vaules obtained were averaged. The two log-fold changes were also averaged to produce a single log FC per gene. The GSE24357 dataset based on the Illumina MouseWG-6 v2.0 Expression Beadchip platform had 12 total samples, of which we considered sham/saline (four samples) as controls and CLP/saline (four samples) as infected ones. GSE15379, based on the Affymetrix Mouse Genome 430 2.0 Array platform, had 12 total samples, of which we considered lung sham wild-type saline (three samples) as controls and lung CLP wildtype (three samples) as infected ones, respectively. 

### 3.2. Meta-Analysis of Sepsis Datasets and DEGs Screening

**Figure 2:**
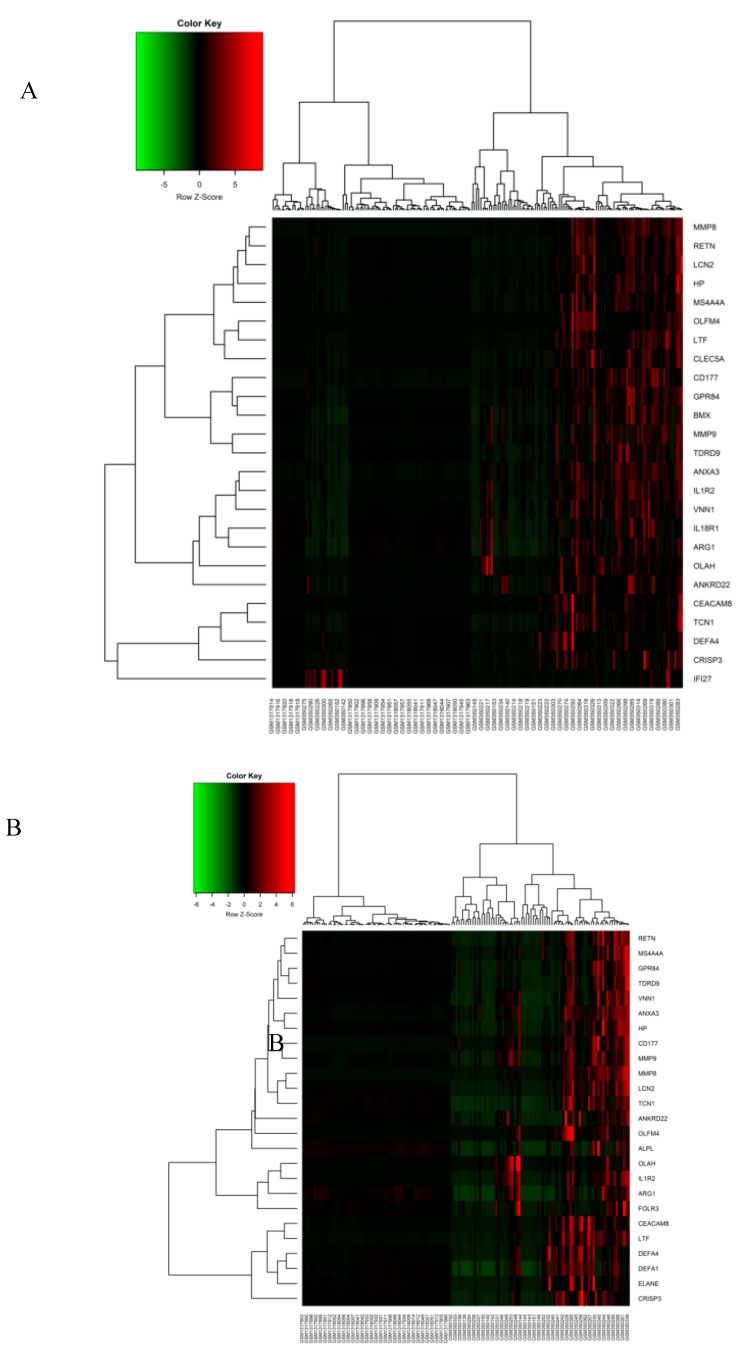
Heatmap for the top 25 upregulated DEGs. Clustering of the top 25 significant DEGs was performed and shown as a heatmap plot in (**A**) the Sepsis day1 group and (**B**) the Sepsis day3 group. A hierarchical clustering algorithm uses an average linkage method and Pearson’s correlation coefficient. Green and red in the plot represent lower and higher expression vaules, respectively.

**Figure 3:**
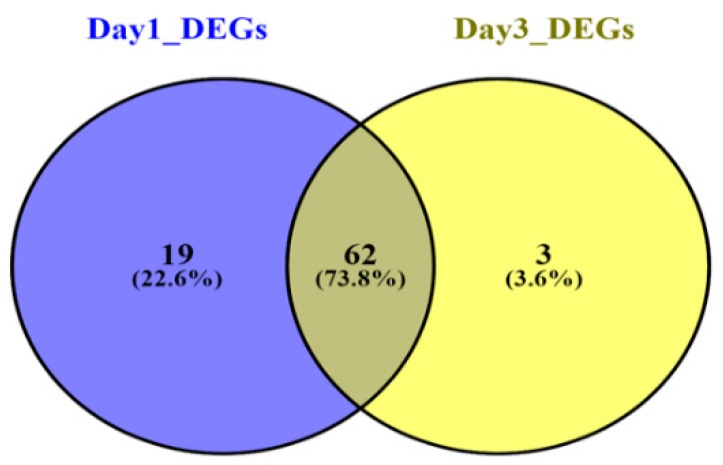
Overlapping DEGs in Sepsis day1 group and Sepsis day3 group. The Venn diagram shows the intersections between the Sepsis day1 group (purple circle) and the Sepsis day3 group (yellow circle). Nineteen genes were included exclusively in “Sepsis day1”, three genes were included exclusively in “Sepsis day3”, and 62 genes were in both groups.

In both human datasets, 146 genes altogether (81 DEGs in Sepsis day1 samples and 65 DEGs in Sepsis day3 samples) were identified as DEGs. DEGs were identified following more than 2.0-fold enrichment (FC, biological significance) over random expectation (*p*-vaule < 0.05 statistical significance). Using the same criteria for screening—BH-corrected *p*-vaule < 0.05 and FC > 2—we discovered that all 146 DEGs were consistently upregulated and there was no gene that was downregulated at this level of significance. However, the day1 sepsis group had highly upregulated genes as compared to the day3 sepsis group. The top 20 upregulated genes in both groups are listed in Table 2. Genes were stacked according to FC, superseded by corresponding *p*-vaule adjustment using the Benjamini‒Hochberg procedure, positioning the FDR. A subset of the top 25 DEGs of both groups was visualized with heatmaps using R and are shown in Figure 2A,B, respectively. Nineteen genes were included exclusively in the “Sepsis day1 group,” three genes were included exclusively in the “Sepsis day3 group,” and 62 genes were common to the “Sepsis day1 group” and “Sepsis day3” groups (Figure 3). From the Venny results, we found that there were 19 DEGs (*BCL2A1*, *C1QB, CEACAM1, CST7, DACH1, DHRS9, FCAR, FGF13, FKBP5, GADD45A, IFI27, IL18RAP, KIF1B, NLRC4, PCOLCE2, PSTPIP2, S100P, SERPINB2, SERPING1*) that were upregulated in sepsis samples on day1, but their expression levels became normal at day3; likewise, there were three genes (CTSG, PI3, VSIG4) that were only upregulated in sepsis samples on day3. Also, a total of 48 DEGs (forty six upregulated and two downregulated) between controls and sepsis-infected CLP samples were filtered out based on the criteria, i.e., *p*-vaules < 0.05 and FC > 2. The lists of sepsis-associated DEGs in human samples and CLP model samples are shown in Supplementary Tables S1 and S2, respectively.

### 3.3. Pathway and Functional Enrichment Analysis

DEGs identified through this meta-analysis approach were classified according to GO hierarchy into functional categories {Cellular Compartment (CC), Biological Process (BP), and Molecular Function (MF)} with a threshold significance of *p*-vaule < 0.05. The most significant sepsis day1 DEGs were enriched in the following descending GO terms: ‘innate immune response’ (GO:0045087), ‘immune response’ (GO:0006955) and ‘defense response to bacterium’ (GO:0042742). ‘Serine-type endopeptidase activity’ (GO:0004252) and ‘extracellular space’ (GO:0005615) were highly enriched GO terms under the MF and CC categories, respectively. The significantly enriched KEGG pathways of sepsis day1 group DEGs were (in descending order): ‘Transcriptional misregulation in cancer’ (hsa05202), ‘*Staphylococcus aureus* infection’ (hsa05150) and ‘Legionellosis’ (hsa05134) (Table 3). On the other hand, the DEGs in the sepsis day3 group were highly enriched for the following GO terms (most significant) under the BP such as ‘innate immune response’ (GO:0045087), ‘defense response to fungus’ (GO:0050832), and ‘defense response to bacterium’ (GO:0042742). The most convincing GO terms under the MF and CC categories were ‘serine-type endopeptidase activity’ (GO:0004252) and ‘extracellular exosome’ (GO:0070062). The significantly enriched KEGG pathways of the sepsis day3 group DEGs were (in descending order) were: ‘Transcriptional misregulation in cancer (has05202), and ‘Amoebiasis’ (hsa05146) (Table 4). From the above analysis, we found that sepsis is closely related to biological processes associated with the immune response. Pathway enrichment analysis of these two groups revealed two common pathways: Transcriptional misregulation in cancer and Amoebiasis. Both these pathways comprised six common functionally enriched DEGs.

### 3.4. PPI Network Analysis

**Figure 4:**
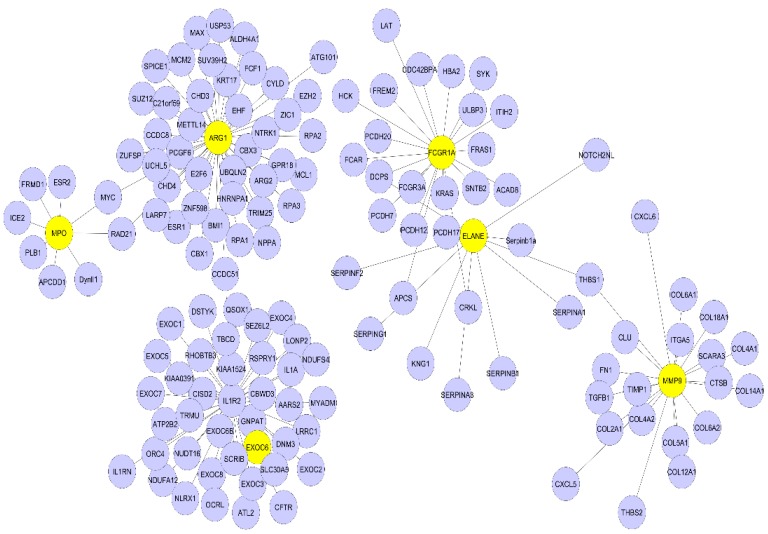
Protein-protein interaction (PPI) network analysis of significantly enriched sepsis-associated DEGs. The network of PPI interaction was constructed from the six identified DEGs. In total, 143 proteins were involved in this network. The six DEGs are in yellow and their interacting partners are purple.

**Figure 5:**
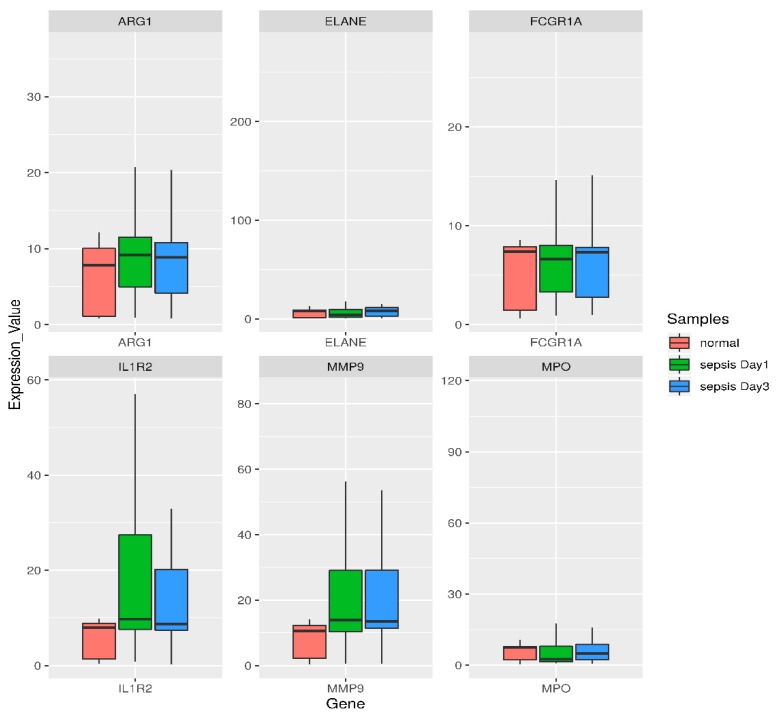
Box- and −whisker plot of six highly upregulated DEGs based on the functional enrichment and PPI analysis for comparing their expression levels. Green corresponds to day1 sepsis, blue to day3 sepsis, and red to control subjects. Genes are shown at the bottom. For individual genes, the vaules of gene expression in log intensities have been normalized to the median of the control group expression. A box plot displays the five-number summary of a set of data: the minimum, first quartile, median, third quartile, and maximum. Endpoints of the axis are labeled by the minimum and maximum vaules. The first and third quartile marks one end and the other end of the box, respectively. The median can be between the first and third quartiles.

To understand the biological meaning of the six upregulated DEGs (*ARG1, IL1R2, FCGR1A*, *MMP9, ELANE, MPO*) identified by the KEGG pathway under the transcriptional misregulation in the cancer pathway and amoebiasis at the protein level, we constructed a PPI network for these six DEGs-encoding proteins with interactions that included 143 nodes and 142 edges, as shown in Figure 4. The topological properties of the PPI network are shown in Table 5. Boxplots comparing the gene expression levels of these six upregulated DEGs are shown in Figure 5. DEGs obtained after the meta-analysis from CLP mice model samples had one gene, i.e., *IL1R2*, in common among the six highly upregulated genes reported in our study. 

### 3.5. Semiquantitative RT-PCR Validation and Immunohistochemistry

**Figure 6:**
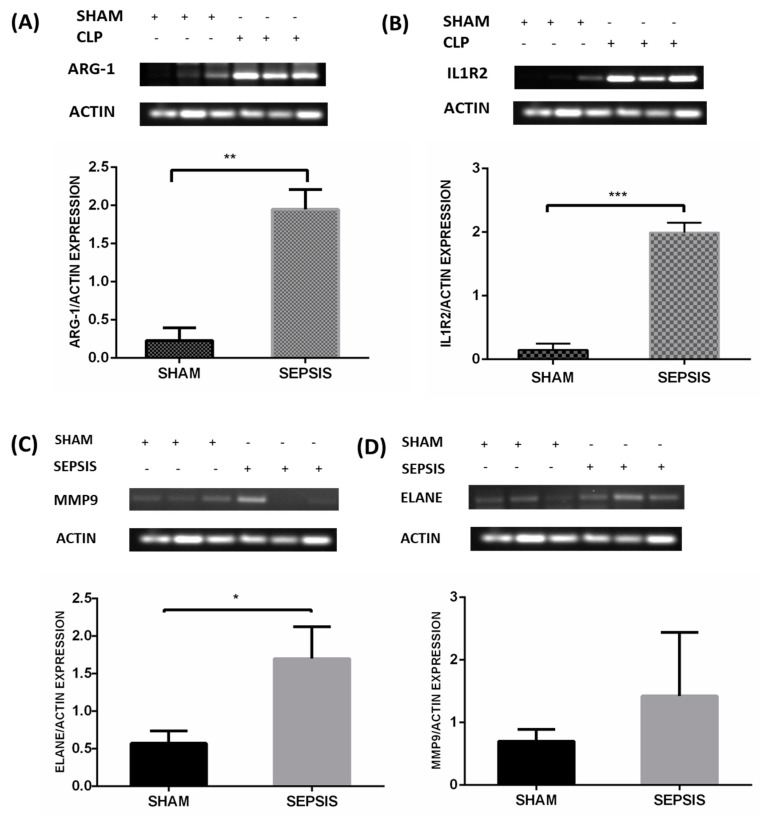
Validation of mRNA expression of selected genes in the lung tissue of an animal sepsis model. Mice were CLP operated for 24 h and then sacrificed; lung tissues were collected for analysis. The figure shows the semi-quantitative mRNA expression and densitometry of (**A**) *ARG1*, (**B**) *IL1R2*, (**C**) *ELANE*, and (**D**) *MMP9* in the lung tissue of the sham and CLP groups. A minimum of three animals were used for each group of animals. Data are presented as mean±SEM, *p* < 0.05. Note: + means treated and − means non-treated.

**Figure 7:**
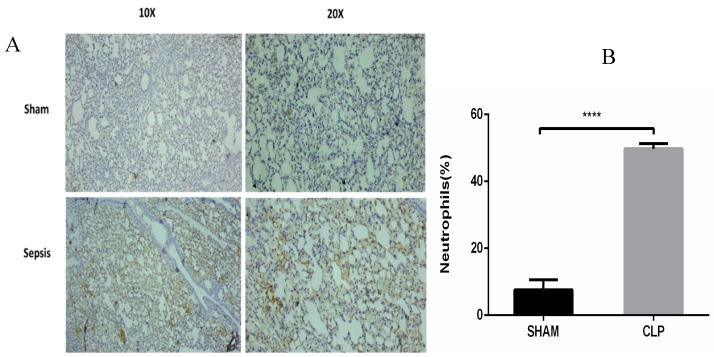
The expression of Myeloperoxidase in neutrophil granulocyte within lung alveoli was assessed by the immunohistochemical technique in the sham and CLP groups. (**A**)This represents strong immunoreactivity in the well-established CLP sepsis animal model as compared to the sham group; (**B**) the significant increase in neutrophil numbers in sepsis as compared to the control. Data are represented as mean ± SEM; experiments were performed in triplicate, with statistical significance at *p*-vaule < 0.0001.

The results were validated in a well-established CLP sepsis animal model. CLP-induced sepsis and sham lung tissues were used to validate the results of the meta-analysis. As per the results of the meta-analysis, the topmost upregulated genes in sepsis, such as *ILIR2, ARG1, ELANE,* and *MMP9*, were validated via semiquantitative RT-PCR as shown in Figure 6A‒D, respectively. Also, the *MPO* expression was assessed by immunohistochemical techniques, as shown in Figure 7A,B.

## 4. Discussion

Sepsis is a substantial cause of morbidity and thus an emerging health concern in pediatrics, geriatric care, and ICUs. A comprehensive study of sepsis’s pathophysiological mechanism will lead us to discover therapies that can elevate the chances of survival. The fundamental component of sepsis pathogenesis is inflammation, which is associated with bacterial infection and dysfunction of the immune system. The lung is one of the organs most often affected in sepsis, mainly because lung infection/pneumonia is often the initial point of the septic process and almost all infections are associated with a systemic inflammatory response (SIRS) in which the lung is the first affected organ [33]. The quest for DEGs has accelerated in recent decades and this differential expression exerts a widespread impact. 

Dataset GSE13904 summarizes the genomic expression profile of critically ill children of sepsis, systemic inflammatory response syndrome, and septic shock; and dataset GSE54514 comprises a transcriptomic analysis of the whole blood of survivors and non survivors of sepsis. In our study, the DEGs of sepsis were identified using a meta-analysis approach and then the topmost functionally significant genes were used for validation in CLP mouse model studies using semi-quantitative RT-PCR and immunohistochemistry. There were 146 differentially upregulated genes obtained from the above-mentioned datasets. The DEGs obtained were remarkably enriched in GO terms under biological processes, the innate immune response, and bacterial and fungal infection. From both the datasets sepsis day1 and day3 samples were compared with normal healthy individuals, and *p*-vaules and fold change were determined for each gene. Genes with *p*-vaule < 0.05 and FC > 2were selected as DEGs. The identified DEGs were further subjected to functional enrichment analysis for understanding their biological implications. GO functional enrichment and KEGG pathway enrichment analysis were carried out using DAVID. DEGs in the sepsis day1 group were enriched in pathways of misregulation in cancer and *Staphylococcus aureus* infection, and sepsis day3 group DEGs were enriched in pathway transcription misregulation in cancer and amoebiasis. The DEGs (*IL1R2, ARG1, FCGR1A, MMP9, ELANE*, and *MPO*) that were common on day1 and day3 of sepsis, with at least 2.0-fold upregulation, were selected for constructing and visualizing the PPI network using Cytoscape. The proteins encoded by these six identified DEGs and their interactions with other proteins were computed from the BioGRID. A total of 143 nodes and 142 edges were identified in the PPI network. The highest degree genes obtained through PPI network analysis were *IL1R2* and *ARG1* (>30), whereas *ELANE, MMP9, FCGR1A, and MPO* were < 30. Using a meta-analysis and network-based approach on samples of sepsis and normal healthy controls, we identified the key genes for inflammation in sepsis with increased expression of *IL1R2* and *ARG1*, as indicated by semiquantitative-PCR studies (Figure 6A,B). We also found a higher expression of *ELANE* and *MMP9* in sepsis. In our expression studies, the results obtained for *ELANE* were significant at *p*-vaule < 0.05, but *MMP9* showed a nonsignificant increase in expression (Figure 6C,D). *MPO* was also overexpressed in sepsis animal tissue samples, as observed by IHC (Figure 7A,B). 

Our study reported that the gene *ARG1* is the topmost upregulated gene in septic patients compared to normal healthy controls, as determined by a PPI network analysis. *ARG1* is a protein-encoding enzyme whose catalytic activity is to hydrolyze arginine to ornithine and urea. Arginase metabolism is a critical regulator of innate and immune responses. A deregulated immune response is one of the major characteristics of sepsis, and *ARG1* metabolism is a regulator of it. The overexpression of *ARG1*, as observed in our results, may play a role in tissue repair. Increased plasma *ARG1* activity depletes the concentrations of L-arginine, the substrate for NO synthesis, leading to vascular dysfunction during severe sepsis and suppressed NO-mediated microbicidal effects [34]. Increased *ARG1* activity may also be a bacterial survival strategy to escape the NO-dependent host antimicrobial immune response [35]. This may also be associated with the M2 macrophage phenotype in sepsis, which is reportedly associated with the wound healing process and tissue repair [36].

The second-most upregulated gene in septic patients was *IL1R2*. Interleukin-1 receptor 2 (*IL1R2*) is responsible for reducing *IL-1* bioavailability by capturing it. Therefore, it acts as an endogenous inhibitor of pro-inflammatory interleukin-1 *(IL1)* signaling [37]. *IL-1* is one of the major pro-inflammatory cytokines that play a critical role in obesity, cancer, heart conditions, and various immune diseases. The activation of endogenous negative regulation of inflammation or the response to anti-inflammatory or immunosuppressive agents has been known to upregulate *IL1R2* expression and soluble *IL1R2* concentrations in biological fluids. Lang et al. [38] reported that *IL1R2* serum concentration is also useful for differentiating between Gram-positive and Gram-negative bacterial infection in sepsis. 

Our study also identified *MMP9, ELANE, and MPO*, which were validated in the CLP model. *MPO* expression was also found to be upregulated, as observed by IHC. Matrix metalloproteins (MMPs) are zinc-dependent endopeptidases that may play a pivotal role in severe sepsis. *MMP9* or gelatinase B, which amounts to slightly more than 0.1% of total bone marrow protein, is thought to be pro-inflammatory [39] and critical to normal vascular development, remodeling, and functioning. This is evidenced by their key functions in processes such as angiogenesis, vasomotortone, and tumor invasion [40]. The results obtained were consistent with studies that suggest a protective role of *MMP9* in sepsis [41]. It is known that basal levels of *MMP9* are highest in the bone marrow. Vandooren et al. [42] reported that, on the induction of endotoxemia, abrupt changes occurred in *MMP9* protein levels, as evidenced by the approximately 90% decrease in protein levels of multimeric *MMP9* and pro*MMP9*. This also coincided with an increase in the (pro) *MMP9* level in the lungs and liver. Similar patterns were observed for the relative expression of the neutrophil markers *ELANE* and *MPO* after the induction of endotoxemia, except in the spleen. *MMP9* is predominantly associated with neutrophils and late stage maturing neutrophils, such as band cells and segmented cells, present in the bone marrow [43]. Myeloperoxidase (*MPO*) and neutrophil elastase (*ELANE*) are the two key neutrophil markers in the blood, liver, spleen, lungs, and bone marrow, as they are most abundantly expressed by neutrophils. Attenuation of sepsis − induced lung injury has been correlated with reduced levels of neutrophil infiltration and chemokine expression by using *MPO* as a marker in several reports [44,45]. As depicted in our results (Figure 7), the increased expression of *MPO* correlates to a higher percentage release of *MPO* by activated neutrophils for antibacterial activities, which causes an increase in degranulated neutrophils [46]. During sepsis, free radical species and *MPO* production exceed antioxidant defenses. This leads to increased oxidative stress, which aggravates inflammation, resulting in direct mitochondrial damage, which leads to major outcomes in sepsis − induced organ dysfunction [47].

However, many studies correlate increased *MMP9* with increased mortality by aggravating severe sepsis [48]. In vivo studies suggest that *MMP9* inhibition or reduction is associated with improved outcomes and increased survival rates in animals [49]. The contradictory results observed with *MMP9* in severe sepsis may be due to differences in the sample population, sampling time, *MMP9* estimation techniques, or clinical endpoints. Our study may point to the important role of *MMP* inhibitors in therapeutic aspects of sepsis. However, *MMP9* inhibition has a limiting therapeutic window. Our study, which pinpointed *ELANE* and *MPO* as important DEGs, may provide insights into targeting neutrophils for the treatment of sepsis to prevent the collateral damage to peripheral organs caused by sepsis.

The results of our study indicate that upregulated *IL1R2* and *ARG1* may be further correlated with the key role of inflammasome in sepsis. Inflammasomes are mediators of secretion of *IL-1* (interleukin-1) family cytokines (e.g., *IL-1β* and *IL-18*) and proteolytic processing. They also cause the release of cell death-related *DAMPs* (damage − associated molecular patterns), e.g., *HMGB1* (high-mobility group box) and *LDH* (lactate dehydrogenase). Pyroptosis, resulting from the excessive activation of inflammasomes, has been implicated in sepsis [50]. Tsalik et al. [51] emphasized the importance of *NLRP3*-inflammasome activation in sepsis survivors, supported by increased expression of the genes downstream from inflammasome activation, including *IL1R2*. Few studies show the role of various drugs that mediate their effects by regulating macrophage polarization and *NLRP3* inflammasome activation [52]. This further indicates that inflammasome assembly*, IL-1 or IL1R2*, macrophage polarization, and the neutrophil recruitment process could be viable drug targets for the treatment of sepsis.

## 5. Conclusions

In conclusion, this comprehensive meta-analysis study of gene expression provides mechanistic insight into sepsis that was further validated in the CLP model. The study demonstrates the role of significant and widespread immune activation, with oxidative stress and the recruitment of neutrophils in sepsis. Our analysis gives a better understanding of the molecular mechanisms associated with sepsis, which may help with choosing plausible targets for designing personalized treatments.

## Figures and Tables

**Table 1:** This table represents the characteristics of discrete studies procured from the Gene Expression Omnibus (GEO) for meta-analysis.

GEO Accession Number	Disease	Sample		Sample Source	Platform
		Day1	Day3		
GSE13904	Sepsis	*n* = 99	*n* = 59	Blood	Affymetrix Human Genome U 133 Plus 2.0 Array
GSE54514	Sepsis	*n* = 35	*n* = 38	Blood	IlluminaHumanHT-12 V3.0 Expression BeadChip

**Table 2:** Top 20 upregulated DEGs in sepsis. The genes were ranked based on the fold change vaule.

Sepsis Day1			Sepsis Day3		
Gene	BH-*p*-Vaule	Fold Change	Gene	BH-*p*-Vaule	Fold Change
*MMP8*	3.03 × 10^−12^	90.83269343	*MMP8*	1.25 × 10^−6^	53.11011783
*OLFM4*	7.66 × 10^−7^	54.94062655	*OLFM4*	0.000177074	33.23986192
*CD177*	1.73 × 10^−15^	27.69570432	*CD177*	2.68 × 10^−7^	24.4262987
*CEACAM8*	2.01 × 10^−6^	17.21435595	*CEACAM8*	1.54 × 10^−6^	18.71820844
*LTF*	3.79 × 10^−11^	14.76500984	*LTF*	1.35 × 10^−7^	14.33448059
*LCN2*	5.64 × 10^−9^	12.69709454	*MMP9*	4.75 × 10^−10^	13.37223858
*OLAH*	3.36 × 10^−9^	12.50259397	*OLAH*	0.000755255	11.87923585
*MMP9*	6.75 × 10^−17^	11.6185382	*LCN2*	8.36 × 10^−7^	10.78809213
*ANXA3*	1.29 × 10^−21^	10.82040432	*DEFA4*	3.74 × 10^−8^	10.37992756
*IL1R2*	2.97 × 10^−19^	10.5634416	*IL1R2*	2.86 × 10^−8^	8.994229615
*RETN*	4.97 × 10^−12^	10.42790653	*ANXA3*	5.44 × 10^−10^	8.817467871
*HP*	8.36 × 10^−13^	10.0624041	*DEFA1*	2.46 × 10^−12^	7.365054135
*GPR48*	4.23 × 10^−15^	8.581561273	*RETN*	9.41 × 10^−7^	6.628552774
*ANKRD22*	2.59 × 10^−14^	7.460137951	*HP*	9.96 × 10^−7^	6.35862726
*CLEC5A*	2.76 × 10^−13^	6.669614066	*MS4A4A*	4.37 × 10^−5^	6.16777804
*DEFA4*	0.000155626	6.278035491	*ELANE*	9.42 × 10^−5^	5.65341365
*MS4A4A*	6.31 × 10^−12^	5.989831378	*VNN1*	3.55 × 10^−8^	5.537816869
*VNN1*	3.55 × 10^−18^	5.855759515	*GPR84*	1.64 × 10^−6^	5.069264307
*TCN1*	3.20 × 10^−10^	5.533269805	*TCN1*	2.72 × 10^−8^	4.837923713

**Table 3:** Sepsis day1 group DEGs’ functional enrichment analysis, representing top GO terms and pathways. Ranking of enriched terms was based on the *p*-vaules.

**GO ID**	**GO Term**	**No. of Genes**	***p*-Vaule**
**Biological Process**			
GO:0045087	innate immune response	17	8.47 × 10^−11^
GO:0006955	immune response	12	3.26 × 10^−6^
GO:0042742	defense response to bacterium	8	4.49 × 10^−6^
**Molecular Functions**			
GO:0004252	serine − type endopeptidase activity	8	1.26 × 10^−4^
GO:0008201	heparin binding	5	0.005346
GO:0004869	cysteine − type endopeptidase inhibitor activity	3	0.009713
**Cellular Component**			
GO:0005615	extracellular space	27	2.49 × 10^−11^
GO:0070062	extracellular exosome	38	2.57 × 10^−11^
GO:0005576	extracellular region	26	6.39 × 10^−9^
**KEGG ID**	**KEGG Pathway**	**No. of Genes**	***p*-Vaule**
**hsa05202**	Transcriptional misregulation in cancer	6	9.47 × 10^−4^
**hsa05150**	*Staphylococcus aureus* infection	4	0.001906
**hsa05134**	Legionellosis	3	0.025598
**hsa05321**	Inflammatory bowel disease (IBD)	3	0.035048
**hsa04610**	Complement and coagulation cascades	3	0.040208
**hsa05146**	Amoebiasis	3	0.085888

**Table 4:** Sepsis day3 group DEGs’ functional enrichment analysis, representing top GO terms and pathways. Ranking of enriched terms was based on the *p*-vaules.

**GO ID**	**GO Term**	**No. of Genes**	***p*-Vaule**
**Biological Process**			
GO:0045087	Innate immune response	14	3.65 × 10^−9^
GO:0050832	Defense response to fungus	5	2.60 × 10^−6^
GO:0042742	Defense response to bacterium	7	1.40 × 10^−5^
**Molecular Functions**			
GO:0004252	Serine − type endopeptidase activity	8	2.29 × 10^−5^
GO:0008201	Heparin binding	5	0.002077
GO:0008233	Peptidase activity	4	0.003483
**Cellular Component**			
GO:0070062	Extracellular exosome	35	8.32 × 10^−13^
GO:0005615	Extracellular space	25	3.64 × 10^−12^
GO:0005576	Extracellular region	21	1.83 × 10^−7^
**KEGG ID**	**KEGG Pathway**	**No. of Genes**	***p*-Vaule**
hsa05202	Transcriptional misregulation in cancer	5	0.001750917
hsa05146	Amoebiasis	3	0.044114518
hsa05322	Systemic lupus erythematosus	3	0.066980623
hsa00052	Galactose metabolism	2	0.091415842
hsa00051	Fructose and mannose metabolism	2	0.097217685

**Table 5:** Topological properties/centrality measures of the PPI network.

Gene	Node Degree	Betweenness	Closeness
***ARG1***	43	0.78	0.49
***IL1R2***	40	0.68	0.44
***FCGR1A***	21	0.63	0.34
***MMP9***	20	0.61	0.33
***ELANE***	10	0.65	0.38
***MPO***	08	0.12	0.27

## Supplementary Materials

The following are available online at https://www.mdpi.com/2073-4425/10/12/1005/s1.

## Data and Materials

The datasets used in our study are available at Gene Expression Omnibus (GEO) under the accession numbers GSE13904, GSE54514, GSE24357, and GSE15379.
